# Splicing junction-based classifier for the detection of abnormal constitutive activation of the KEAP1-NRF2 system

**DOI:** 10.1038/s41540-024-00475-w

**Published:** 2024-12-06

**Authors:** Raúl N. Mateos, Wira Winardi, Kenichi Chiba, Ai Okada, Ayako Suzuki, Yoichiro Mitsuishi, Yuichi Shiraishi

**Affiliations:** 1 Division of Genome Analysis Platform Development, National Cancer Center Research Institute, 5-1-1 Tsukiji, Chuo-ku, Tokyo 104-0045 Japan; 2 Division of Respiratory Medicine, Juntendo University Graduate School of Medicine, 2-1-1 Hongo, Bunkyo-ku, Tokyo 113-8421 Japan; 3 Department of Computational Biology and Medical Sciences, Graduate School of Frontier Sciences, The University of Tokyo, 5-1-5 Kashiwanoha, Kashiwa, Chiba 277-8561 Japan

**Keywords:** Cancer, Computational biology and bioinformatics

## Abstract

The KEAP1-NRF2 system plays a crucial role in responding to oxidative and electrophilic stress. Its dysregulation can cause the overexpression of downstream genes, a known cancer hallmark. Understanding and detecting abnormal KEAP1-NRF2 activity is essential for understanding disease mechanisms and identifying therapeutic targets. This study presents an approach that analyzes splicing patterns by a naive Bayes-based classifier to identify constitutive activation of the KEAP1-NRF2 system, focusing on the higher presence of abnormal splicing junctions as a subproduct of overexpression of downstream genes. Our splicing-based classifier demonstrated robust performance, reliably identifying activation of the KEAP1-NRF2 pathway across extensive datasets, including The Cancer Genome Atlas and the Sequence Read Archive. This shows the classifier’s potential to analyze hundreds of thousands of transcriptomes, highlighting its utility in broad-scale genomic studies and provides a new perspective on utilizing splicing aberrations caused by overexpression as diagnostic markers, offering potential improvements in diagnosis and treatment strategies.

## Introduction

Gene expression regulation is a dynamic process that involves a complex interplay among various key components, including transcription factors, RNA splicing machinery, and epigenetic modifications, ensuring the appropriate production of proteins and the maintenance of cellular homeostasis^1,2^. One system that plays a crucial role in gene expression regulation is the KEAP1-NRF2 pathway^3^. The molecular dynamics of this system are as follows: under normal conditions, a KEAP1 dimer binds to the NRF2 protein (encoded by the *NFE2L2* gene) by the DLG and ETGE motifs within the Neh2 domain located in exon 2. This interaction targets NRF2 for ubiquitination by the CUL3 complex, ultimately leading to its degradation via the proteasome^4,5^. This frees the KEAP1 molecules and allows them to interact with new NRF2 molecules, closing the cycle. However, when cells are exposed to oxidative and electrophilic insults, KEAP1 undergoes conformational changes that prevent a proper interaction with NRF2, leading to the stabilization and nuclear translocation of NRF2^3,6–8^. Once in the nucleus, NRF2 binds to the antioxidant response elements (ARE) in the promoter regions of its target genes and activates their expression, promoting cellular detoxification^9^.

Multiple inducers can activate the KEAP1-NRF2 pathway, including endogenous signaling metabolites and external factors like UVA radiation and dietary components^10,11^. Notably, mutations in the KEAP1-NRF2 system, often seen in cancer, result in an abnormal constitutive activation of the pathway, leading to overexpression of downstream genes which confers resistance to chemotherapy through protection towards oxidative and electrophilic stress, causing the cancer cells to acquire malignancy and increased survival^10,12–17^. Recent progress in deciphering this pathway has paved the way for novel therapeutic approaches, which have demonstrated varied results^18,19^. Therefore, detection of abnormal constitutive activity of the KEAP1-NRF2 pathway is crucial for a rapid medical response and targeted therapies.

In order to explore the detection of abnormal activity of the KEAP1-NRF2 system, several methodologies primarily focus on measuring the pathway’s activity through the expression levels of downstream genes^20,21^. However, this gene expression-based approach faces several challenges. One significant issue is the potential bias in the genes selected as markers of pathway activity, which can vary depending on the tissue type and specific cancer. Many approaches fail to consider the dynamic nature of cellular responses at the pathway level, which is crucial for understanding biological variation over time^22^. Not recognizing the biological context and the variability of these markers could render them ineffective on different tissues or samples^23^. Furthermore, these methods often overlook critical aspects of biological complexity. For example, the presence of multifunctional genes and the hierarchical parent-child relationships among some genes in these marker lists are frequently ignored, reducing the accuracy and robustness of these detection strategies^24^. Additionally, accurately quantifying absolute gene expression values, such as using housekeeping genes for reference, poses substantial difficulties. These methods rely on the assumption that housekeeping genes are consistently expressed across different tissues, which does not consistently hold across all contexts^25–27^. Finally, these methods typically require a cohort for comparison, limiting their application to individual samples as they rely on relative activity levels within a group and need normalization.

**Fig. 1:**
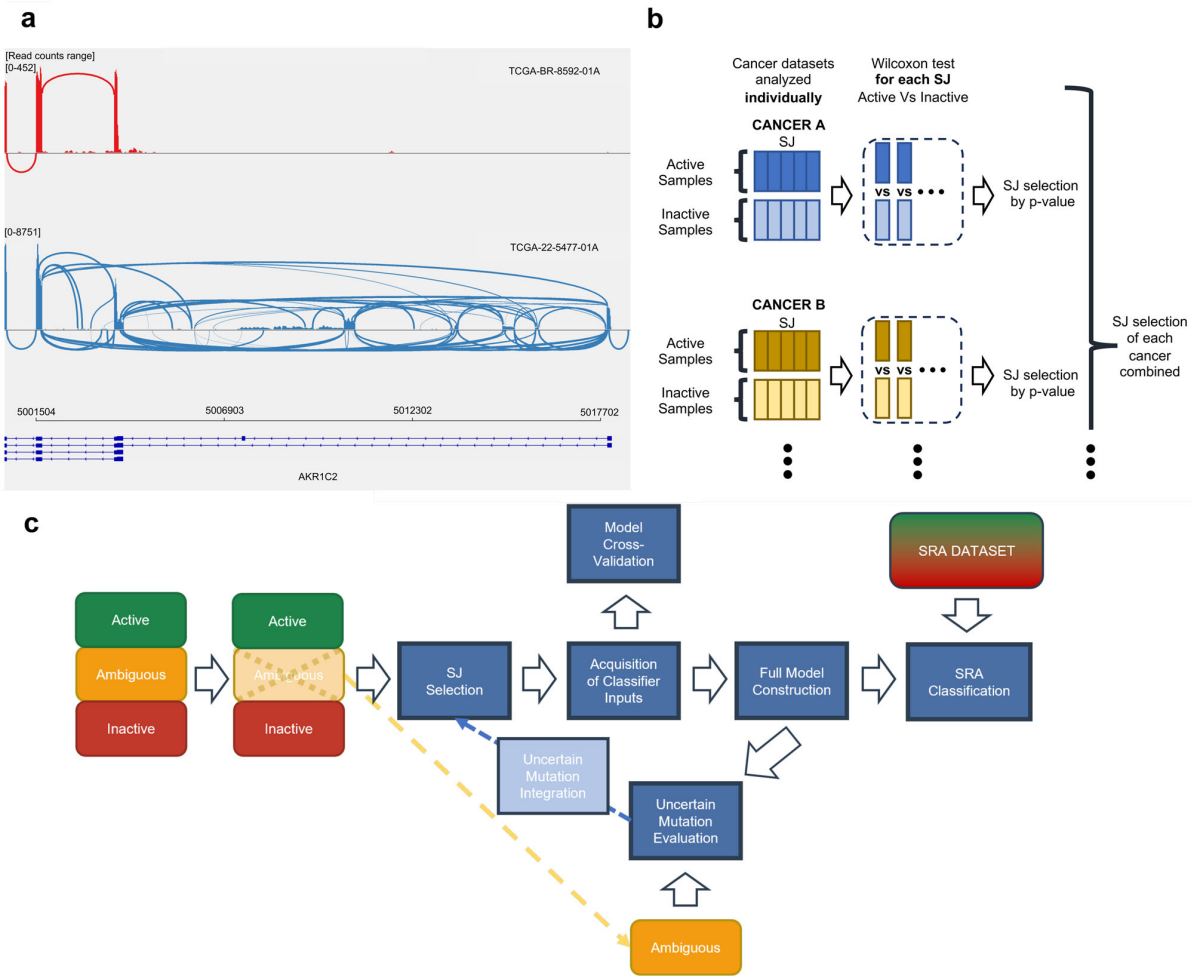
Concept and design. **a** Differential SJ Patterns due to *AKR1C2* overexpression by constitutive activation of the KEAP1-NRF2 System. Sashimi plot illustrating the impact of *AKR1C2* overexpression on SJ dynamics. The genomic coordinates are displayed along the x-axis, while the y-axis indicates the read counts. The thickness of the lines represents the number of reads supporting the junction. The red section represents a control sample with no KEAP1-NRF2 system alterations, exhibiting a standard junction pattern. Contrastingly, the sample affected by KEAP1-NRF2 system disturbance shows both a pronounced *AKR1C2* overexpression, evidenced by increased SJ counts, and the presence of abnormal SJ at previously unannotated locations. **b** Structure of the pipeline. Our model is first designed using only Active and Inactive samples, setting Uncertain samples aside for posterior analysis. From this dataset, abnormal SJs and their corresponding normal SJs were selected as the input required for the construction and evaluation of the model generated. With the model built, we evaluated and integrated the Uncertain mutations, leading to the rebuild of the model with an updated abnormal SJ pair selection. An initial cross-validation analysis was performed, and then the complete model using the relabeled dataset was generated and implemented on the SRA dataset. **c** Abnormal SJ selection. For each cancer type, abnormal SJs were evaluated using a one-tailed Wilcoxon test to compare their presence between Active and Inactive samples. The *p*-values were adjusted using the Bonferroni method. Abnormal SJs with adjusted *p*-value ≤ 0.001 were selected from each cancer type and compiled as inputs for our classifier.

Instead, we have shifted our focus to the splicing machinery, aiming to approach the detection of constitutive activation of the KEAP1-NRF2 pathway often caused by mutations on the system from a perspective that could potentially overcome the tissue-specific and sample normalization challenges associated with current gene expression-based methods. Cellular RNA splicing under normal conditions ensures the removal of erroneous transcripts^28^, but this process is compromised during overexpression, a common result of mutations in pathways like KEAP1-NRF2. This dysregulation leads to an overwhelmed splicing system and an increase in aberrant transcripts (Fig. 1a). Our research aims to use the abnormal splicing junctions (SJs) —coordinates where RNA is cut and rejoined to remove non-coding regions and concatenate coding sequences—of these aberrant transcripts as markers to identify constitutive activation of the KEAP1-NRF2 pathway, distinguishing normal from overexpressed cellular states.

In this study, we present an approach to predict consistent activation of the KEAP1-NRF2 pathway based on abnormal SJs resulting from gene overexpression caused by dysregulation-inducing mutations. We employed a naive Bayes-based model to infer the presence of mutations affecting the pathway through the analysis of abnormal SJ patterns in samples. We validated our method using The Cancer Genome Atlas (TCGA) dataset consisting of patients with diverse genetic disorders. By comparing our approach with a naive approach using the expression data from the same samples, we demonstrated that our classifier can match gene expression-based classifiers in identifying the constitutive activation of the pathway.

## Results

### Classifier overview

To construct a classifier for predicting the abnormal activation of the KEAP1-NRF2 system, we used the TCGA database. This database provides comprehensive information on mutations related to the KEAP1-NRF2 pathway and allows for the acquisition of SJ information. We categorized KEAP1-NRF2 pathway related mutations found in the TCGA database into three classes; “Functional,” “Uncertain,” and “Non-Functional” based on the annotation of their effect on the KEAP1-NRF2 pathway. For the construction of the classifier, we used samples labeled as “Active” (having at least a Functional mutation) and “Inactive” (Non-Functional mutations or no mutations in the KEAP1-NRF2 system) based on their mutation status. Samples with only Uncertain mutations, labeled as “Ambiguous” samples, were left out from this step for further evaluation. We constructed our classifier using a naive Bayes approach. In order to account for the overdispersion observed in aberrant SJ distributions, our classifier was coupled with beta-binomial distributions, where the beta parameters of the model address this overdispersion by allowing for variability beyond sampling noise^29,30^. More specifically, the procedure is as follows (Fig. 1b, c and Methods for details):

1. We retrieved SJ counts using recount3^31^. Then, we selected abnormal SJs and their corresponding normal SJs sharing coordinates with the abnormal SJs (which we defined as “abnormal SJ pairs”). The selection was performed based on the presence of a significantly higher count of abnormal SJs in Active samples versus Inactive samples.
2. Assuming the generative model of the counts of abnormal SJ pairs follows a beta-binomial distribution, we fit beta-binomial distributions for each pair of SJ separately for the sets of Active and Inactive samples.
3. Then, for new samples (and their new set of SJ counts), we can calculate the generative probabilities for both Active and Inactive cases as the ratio of the beta-binomial probabilities. The logarithm of the ratio of the probabilities is defined as the NRF2 score (see Methods for details):

1 $$\begin{array}{l}{\rm{NRF}}2\,{\rm{Score}}={\mathrm{ln}}\left(\frac{{\rm{P}}({\rm{Active}})}{{\rm{P}}({\rm{Inactive}})}\right)\\\qquad\qquad\qquad ={\mathrm{ln}}({\rm{P}}({\rm{Active}}))-\mathrm{ln}({\rm{P}}({\rm{Inactive}}))\end{array}$$

### Mutation categorization

We compiled a list of several classes of somatic genomic variants affecting the *KEAP1*, *NFE2L2*, and *CUL3* genes from 9533 TCGA samples including single nucleotide substitutions, insertions, deletions, copy number alterations, and an RNA alteration (exon skipping) that potentially activates the KEAP1-NRF2 pathway. We classified these alterations into three classes: Functional, Uncertain and Non-Functional, based on their location, statistical recurrence and potential effects as reported in scientific literature^32^.

- Functional mutations: Oncogenic and Likely Oncogenic somatic mutations found in *KEAP1*^33^ (95 samples) as well as the somatic mutations occurring at *NFE2L2* exon 2 hotspot locations^15,34^ (184 samples). We adopted the annotation of oncogenicity and hotspot as defined on cBioPortal (see Methods).
- Uncertain mutations: Comprises mutations whose effects on the pathway are not as extensively investigated as in the case of the previous mutations and require further exploration. This set consists of somatic mutations in *KEAP1* not labeled as Oncogenic or Likely-Oncogenic (159 samples), somatic mutations in *NFE2L2* on non-hotspot locations of exon 2 (13 samples), somatic mutations in *NFE2L2* on exons other than exon 2 (50 samples), somatic mutations in *CUL3* (146 samples), copy number amplifications (CNA) in *NFE2L2* (100 samples), and exon 2 skipping instances in *NFE2L2* including the reported case of exon 2 + 3 skipping^35^ (29 samples).
- Non-Functional mutations: Mutations that did not fall into any of the previous categories such as 3’ UTR, 5’UTR, intron and silent mutations.

Summary of the labeling is provided in Table 1. Details of the mutation categorization are provided in the Methods section.

**Table 1:** Summary and description of the mutation labeling performed in this study

Mutation	Functionality label	Samples	Functionality label after evaluation	Description
*KEAP1* Oncogenic	Functional	95	Functional	Oncogenic Somatic mutations in *KEAP1*
*NFE2L2* Exon 2 Hotspot	Functional	184	Functional	Somatic mutations at hotspots of *NFE2L2* reported as Oncogenic or Likely Oncogenic by OncoKB
*CUL3* Mut	Uncertain	146	Uncertain	Somatic mutations in *CUL3*
*KEAP1* non-Oncogenic	Uncertain	159	Functional	Somatic mutations in *KEAP1* not reported as Oncogenic or Likely Oncogenic by OncoKB
*NFE2L2* CNA	Uncertain	100	Uncertain	Copy number amplification of *NFE2L2*
*NFE2L2* Exon 2 non-Hotspot	Uncertain	13	Non-Functional	Somatic mutations at exon 2 of *NFE2L2* not reported as Oncogenic or Likely Oncogenic by OncoKB
*NFE2L2* Exon 2 Skipping	Uncertain	29	Functional	Presence of at least 2 reads supporting the skipping of exon 2 or exon 2 + 3 of *NFE2L2*
*NFE2L2* Other Exon Mut	Uncertain	50	Uncertain	Somatic mutations in *NFE2L2* at exons other than exon 2
None	Non-Functional	8757	Non-Functional	None of the previous mutations is present in the sample

The table includes a column with the labeling after the evaluation and reintegration of Uncertain mutations.

### Sample categorization

With the mutation categorizations in place, we were able to label the samples based on their KEAP1-NRF2 system mutation status:

- Active: Samples with Functional mutations, which are expected to possess a constitutive activation of the KEAP1-NRF2 system (279 samples).
- Ambiguous: Samples without any Functional but with Uncertain mutations (497 samples).
- Inactive: Samples without any Functional or Uncertain mutations, expected to lack constitutive activation of the KEAP1-NRF2 pathway (8757 samples).

### Evaluation of uncertain mutation classes

To assess whether Uncertain mutations should be classified as Active or Inactive, we examined the NRF2 scores of Ambiguous samples generated by our classifier constructed from Active and Inactive sample data. We extracted abnormal SJ pairs where the count of abnormal SJs was significantly higher in Active samples compared to Inactive ones for 33 distinct cancer type cohorts separately. Then, by compiling the abnormal SJ pairs from every cancer type, we obtained a collection of 1623 abnormal SJ pairs.

Next, to evaluate the impact of each Uncertain mutation on the KEAP1-NRF2 pathway activity, we calculated the proportion of samples with NRF2 scores exceeding the threshold of 10 (see Methods). Mutation classes with more than 40% samples scoring above the set threshold were relabeled as Functional. Classes where 10–40% of the samples had NRF2 scores surpassing the threshold retained their Uncertain designation and were subsequently excluded from further analysis. Finally, classes where fewer than 10% of the samples surpassed the threshold were redefined as Non-Functional.

**Fig. 2:**
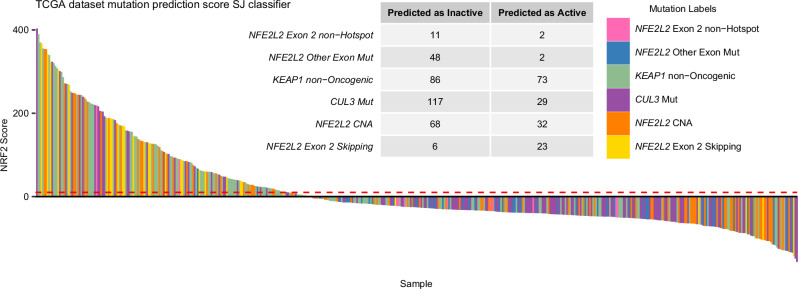
Uncertain mutations. Evaluation of Uncertain mutations. Bar plot of the NRF2 scores obtained for every Ambiguous sample. The table shows the number of samples the model classified as Active or Inactive, which we used to reintegrate the mutations into the model. The NRF2 score threshold, set at 10, is indicated in red.

The observation of elevated NRF2 scores in 73 out of 159 samples with *KEAP1* mutations not defined as Oncogenic or Likely-Oncogenic (as defined on cBioPortal) (45.91%) and in 23 out of 29 samples exhibiting *NFE2L2* exon 2 skipping alterations (79.31%) led to the reclassification of these Ambiguous labels as Functional. In contrast, *CUL3* mutation (29/146, 19.86%), *NFE2L2* CNA (32/100, 32.00%), *NFE2L2* exon 2 mutations outside of hotspots (2/13, 13.33%) were considered inconclusive, remaining as Uncertain. Only 2 out of 50 samples with *NFE2L2* mutations in other exons (4.00%) passed the threshold, leading to the reclassification of this mutation as Non-Functional, indicating that it likely does not influence the activity of the pathway (see Methods, Fig. 2). The number of Active, Ambiguous, and Inactive samples were updated to 467, 259, and 8807 respectively. The reclassification led to all *KEAP1* mutations being deemed Functional in our model. Yet, the oncogenic impact of many such mutations remains undetermined or unreported on cBioPortal. Consequently, this can result in low NRF2 scores in samples where the oncogenic effect of their *KEAP1* mutation is not confirmed.

### Evaluation of NRF2 score on TCGA samples

Based on the updated mutation and sample labels, we repeated the selection of abnormal SJ pairs. This refined approach yielded a list of 1705 abnormal SJ pairs.

**Fig. 3:**
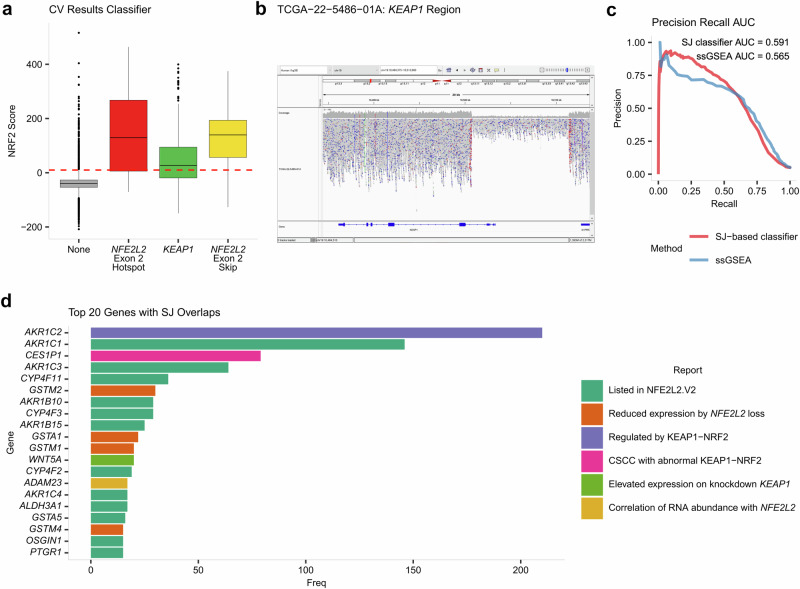
Model evaluation and abnormal SJ genomic location. **a** Boxplot of the NRF2 scores obtained during cross-validation of the model. **b** IGV visualization of the *KEAP1* deletion occurring in TCGA-22-5486-01A, an alteration detected outside the mutations predefined in the scope of our study. **c** Precision-Recall curve comparing the ssGSEA method (in blue) with the SJ-based classifier (in red) with AUC values used for comparing both methods. **d** Gene overlap of the selection of abnormal SJs. Bar graph depicting the top 20 genes ranked by the frequency of overlap with selected abnormal SJs for our model. All the genes in the top 20 are associated with the KEAP1-NRF2 system, indicated by color.

We then conducted a two-fold cross-validation analysis with a new selection of abnormal SJ pairs, splitting the Active samples and Inactive samples in half for training and testing the model while assuming that Active and Inactive samples are positive and negative cases, respectively. Setting the threshold of our classifier at 10 resulted in identifying 8534 Inactive samples scoring below the threshold and 273 scoring above it. Among Active samples, 305 met the threshold, while 162 did not. These results produced a specificity of 96.90%, a precision of 52.77% and a recall of 65.31% (Fig. 3a). Given the considerable imbalance between Inactive and Active samples in the dataset, these results show the challenge of this classification and reflect the robust performance of the classifier under challenging conditions.

Although our classifier effectively identified samples with abnormal activation of the KEAP1-NRF2 pathway, 273 out of 8534 Inactive samples got assigned NRF2 scores of at least 10. The occurrence of high NRF2 scores on Inactive samples suggests the presence of previously undetected alterations. To further investigate this possibility, we explored the copy number information of Inactive samples with scores surpassing the classifier threshold. We extracted 15 samples with signs of potential copy number alterations in *KEAP1* and *NFE2L2* regions and with available whole-genome sequencing (WGS) data (see Methods for details, Supplementary Table 1).

Among the 15 samples analyzed, 3 exhibited clear structural variations upon manual evaluation of whole-genome sequencing data. For TCGA-22-5485-01A (NRF2 score of 516.12), a deletion of the first exon and promoter region of *KEAP1* was identified (Fig. 3b). Similarly, TCGA-78-7150-01A (NRF2 score of 60.28) displayed a deletion spanning the entire *KEAP1* region (Supplementary Fig. 1). TCGA-DD-AACZ-01A (NRF2 score of 247.35) showed a deletion downstream of *KEAP1* (Supplementary Fig. 2), which is likely to disrupt its function, potentially explaining the observed abnormal pathway activity indicated by the elevated NRF2 score. The identification of deletions in these three cases, originally classified as Inactive, reveals previously undetected genetic modifications. This suggests that more in-depth genomic profiling is essential to detect such critical changes and to refine our understanding of the pathway’s activity in different cancer types.

### Comparison with other approaches

In order to evaluate the performance of our method, we conducted a parallel analysis utilizing single-sample Gene Set Enrichment Analysis (ssGSEA)^36^ an extension of the well-established Gene Set Enrichment Analysis (GSEA) technique^37^. Unlike GSEA, which compares enrichment scores across groups of samples for specific gene sets, ssGSEA calculates a unique enrichment score for each individual sample. In this study, we applied ssGSEA to analyze a set of NRF2 target genes (see Methods). The resulting scores were then interpreted as indicators of NRF2 activation and subsequently used to compare the discriminative accuracy of this approach with that of the SJ-based method.

Precision-Recall curves were generated to compare our SJ-based classifier with the ssGSEA method. The area under the curve (PR-AUC) served as our metric for evaluation. The SJ-based classifier demonstrated a performance with a PR-AUC of 0.59, whereas the ssGSEA analysis obtained a PR-AUC of 0.56. ssGSEA’s inability to normalize on individual samples limits its use, since it cannot establish a universal cutoff for classification. Conversely, our SJ-based classifier leverages pairs of altered and normal SJs for internal normalization within each sample. Since the NRF2 score is a log ratio of probabilities, the SJ-based classifier’s natural threshold is zero (see Methods), which facilitates its application across independent samples. This inherent threshold, absent in the ssGSEA method, makes our SJ-based classifier more suitable for detecting constitutive activation in the KEAP1-NRF2 system (Fig. 3c). Additionally, the ssGSEA score is limited to the expression levels of the selected genes (in this case, genes associated with NRF2 transcription factor targets), which may vary across different tissues and cancer types. This variability reduces the flexibility of the ssGSEA approach, whereas the SJ-based classifier is better suited to handle diverse datasets, both in quantity and type.

### SJ genomic location analysis

We proceeded to investigate the specific genomic locations of the abnormal SJs used by our classifier, particularly whether they overlap with downstream genes of the KEAP1-NRF2 system. Our analysis found that the selection of 1705 abnormal SJs overlapped with 452 coding genes. Notably, among the genes overlapping with the abnormal SJ set, the top 20 most frequently overlapped genes were predominantly genes associated with the KEAP1-NRF2 system (Fig. 3d). Specifically, 12 of these top 20 genes (*AKR1C1*, *AKR1C3, CYP4F11, AKR1B10, CYP4F3*, *AKR1B15*, *CYP4F2*, *AKR1C4*, *ALDH3A1*, *GSTA5, OSGIN1* and *PTGR1)* were found to be NRF2-activated as listed in the Human Gene Set: NFE2L2.V2 from https://www.gsea-msigdb.org/. Further examination of the remaining 8 genes revealed their linkage to the pathway: *GSTA1*, *GSTM1*, *GSTM2*, and *GSTM4* were identified as experiencing reduced expression in *NFE2L2* knockout mice^38^. *CES1P1* has been reported as highly expressed in cervical squamous cell carcinoma (CSCC) patients with abnormal KEAP1-NRF2 system activity, while also showing potential as a prognostic biomarker^39^. Another study reported *AKR1C2*, among other AKR genes, as being regulated by the KEAP1-NRF2 system^40^. Elevated expression of *WNT5A* following *KEAP1* knockdown or deletion was also observed^41^. Although no reported association of *ADAM23* expression with the KEAP1-NRF2 system was found, LinkedOmicsKB analysis (https://kb.linkedomics.org/) shows a significant correlation (Spearman correlation *p*-value ≤ 0.001) in RNA abundance between *NFE2L2* and *ADAM23* in BRCA, HNSC and LSCC. These results align with our predictions, as activation of the pathway typically elevates the expression of downstream genes. Consequently, this overexpression leads to a greater prevalence of abnormal SJs in these genes compared to others not influenced by the KEAP1-NRF2 system.

### SRA database analysis: overview

Having confirmed the efficacy of our method on the TCGA dataset, we generated the final classifier. We trained it using the complete set of 9274 Active and Inactive samples, and then applied it to the project compilation provided by the Sequence Read Archive (SRA). The SJ information of the 8592 SRA projects, comprised of 283,132 runs, were downloaded using the R package recount3. Overall, our classifier retrieved 886 runs with a score of at least 10, belonging to 228 different projects.

**Fig. 4:**
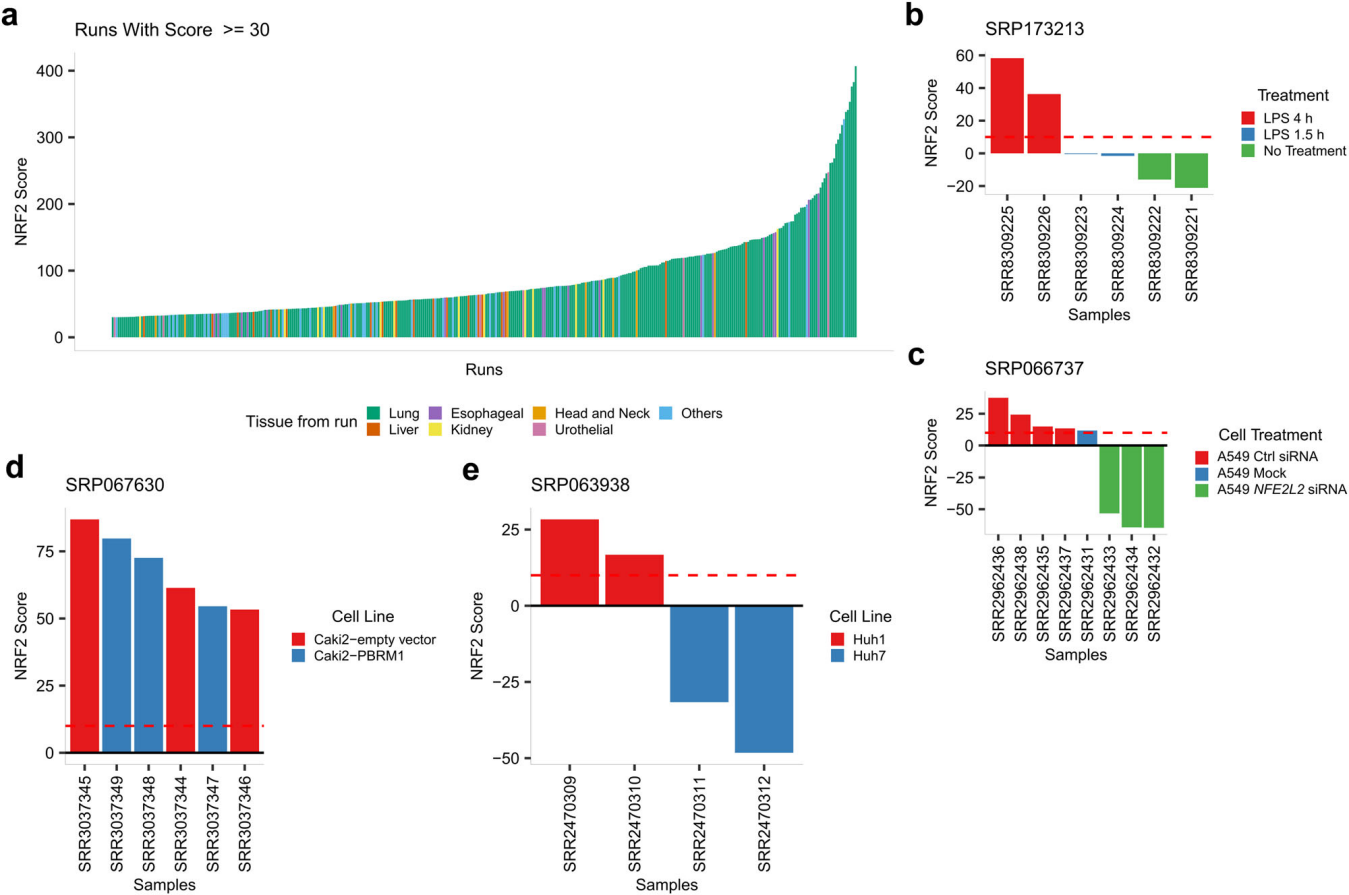
SRA analysis. **a** SRA Overview: Exploration of runs with NRF2 score of at least 30 and their associated tissue of study. Runs are plotted by NRF2 score and colored by the tissue analyzed. **b** Results of our classifier on project SRP173213. The NRF2 score increases with the duration of LPS treatment in monocytes, reflecting the NRF2-mediated activation observed during LPS-induced inflammatory responses in monocytes. **c** Results of our classifier on project SRP066737. Our classifier successfully differentiated between A549 with and without *NFE2L2* knockdown. **d** Results of our classifier on project SRP067630. The Caki-2 cell line was predicted to have constitutive activation across the study. **e** Results of our classifier on project SRP063938. Our classifier was capable of distinguishing between different *KEAP1* mutations: the missense mutation in Huh1 and the silent mutation in Huh7.

We explored the SRA projects to assess the efficacy of our classifier in identifying high NRF2 scores in KEAP1-NRF2 related projects. The threshold was strengthened, focusing on samples with a minimum NRF2 score of 30, which retrieved 381 runs. Out of these 381 runs surpassing this threshold, 273 (71.65%) belonged to lung runs, which corresponded with our expectations, as alterations in the KEAP1-NRF2 pathway are predominantly observed in LUAD and LUSC^8^. Additionally, 20 runs belonged to liver (5.25%), 19 to esophagus (4.99%), 13 to kidney (3.41%), 10 to head and neck (2.62%) and 6 to urothelial (1.57%). The remaining 40 runs (10.50%) belonged to other less frequent tissues and projects (Fig. 4a).

While most of these high-scoring runs belonged to projects that focused on cancer, we also found 4 high-scoring runs belonging to three distinct projects not associated with cancer. In the SRP173213 project, healthy donor monocytes were treated with or without LPS for 1.5 and 4 h. The NRF2 scores obtained from our classifier increased over time and exceeded the threshold after 4 h of LPS exposure, consistent with NRF2-mediated activation in LPS-induced inflammatory responses in monocytes^42,43^ (Fig. 4b). SRP126155 assessed the impact of cigarette smoke on nasal epithelial cells, where one sample notably scored 41.76. However, this sample was part of a larger set of technical replicates, suggesting other factors might influence this high score rather than smoke exposure alone (Supplementary Fig. 3). Lastly, in SRP151606, which explored the sexual dimorphism of preeclampsia-dysregulated transcriptomic profiles and the endothelial function in fetal endothelial cells, two samples exceeded an NRF2 score of 30, but insufficient project details left their link to the KEAP1-NRF2 system unresolved.

### SRA database analysis: exploration of KEAP1-NRF2 related cell line projects

We conducted a more scrutinous analysis on specific projects involving cell lines known to have alterations in the KEAP1-NRF2 system. The project SRP066737 studied the activation of lncRNAs downstream of the KEAP1-NRF2 pathway using the cell line A549, known for having a missense variant in *KEAP1* (p.G333C), mutation labeled as Oncogenic on cBioPortal. For that purpose, they compared three samples transfected with siRNA targeting *NFE2L2* against four control samples. Our classifier successfully differentiated the transfected samples, assigning scores above the threshold to cell lines not treated with siRNA and negative scores to the siRNA-treated samples (Fig. 4c).

Using as reference the findings on NRF2 activation reported by Taguchi et al.^10^ we also examined additional cell lines with abnormal activation of the KEAP1-NRF2 system from the SRA dataset. Among these cell lines is Caki-2, a clear cell renal carcinoma line with accumulation of the protein p62. This protein, coded by the SQSTM1 gene, functions as an autophagosome cargo protein, targeting proteins that bind to it for selective autophagy. The accumulation of p62 has been reported to cause the activation of the KEAP1-NRF2 pathway^44^. Used in the project SRP067630, the Caki-2 cell line received a high NRF2 score in every sample explored (Fig. 4d). Another interesting result was found in the SRP063938 project, where two hepatocellular carcinoma cell lines with *KEAP1* mutations were used: Huh1, which has a missense mutation (p.N414Y), and Huh7, which has a silent mutation (p.Y334Y). Despite no reported oncogenicity for these mutations on cBioPortal, the high NRF2 scores for Huh1 indicates a potential activation of the pathway by the missense mutation, while the negative scores for Huh7 hint at the lack of impact of the silent mutation (Fig. 4e).

**Fig. 5:**
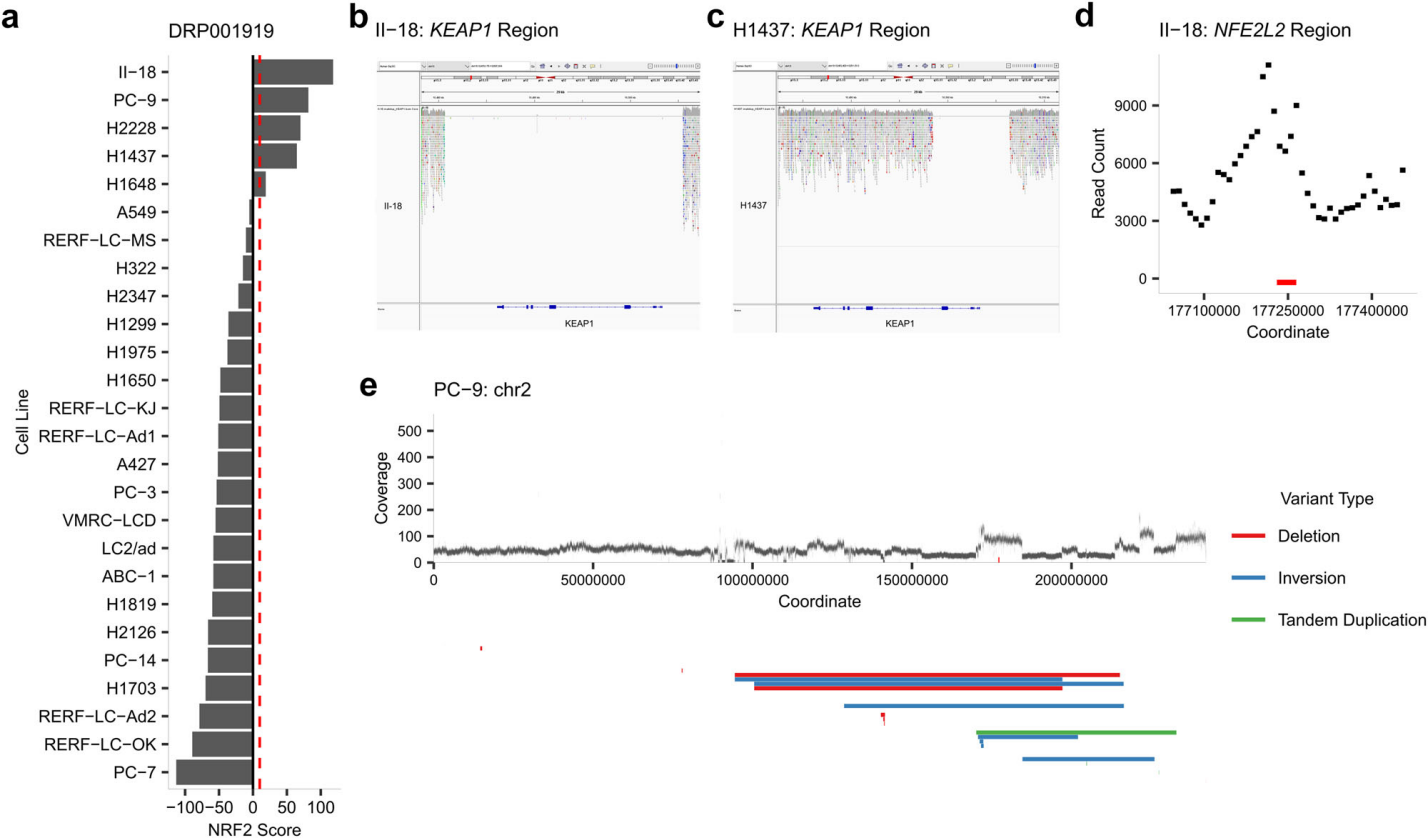
Project DRP001919 analysis. **a** Results of our classifier on project DRP001919. **b** IGV visualization of the *KEAP1* deletion occurring in the cell line II-18, previously unreported. **c** Similarly, IGV visualization of the *KEAP1* region of the cell line H1437 presents a previously unreported deletion. **d** Coverage exploration of the *NFE2L2* region in II-18, showing signs of amplification affecting *NFE2L2* (coordinates of the gene marked in red). **e** Coverage exploration across chromosome 2 of cell line PC-9. Coverage analysis as well as structural variation analysis revealed a complex structural variation across the q arm of chromosome 2 impacting *NFE2L2*.

Another project analyzed, DRP001919, performed a multi-omics catalogue of 26 lung adenocarcinoma cell lines (Fig. 5a), six of which harbor known mutations in the KEAP1-NRF2 system. The cell line H2228 possesses a missense variant in *NFE2L2* reported on cBioPortal at the hotspot location p.G31A. Accordingly, the classifier assigned an NRF2 score of 70.07. H1648, which has a missense variant not reported on cBioPortal in *KEAP1* at p.G364C, obtained a score of 16.86, also surpassing our threshold. The cell line A549, with a loss-of-function mutation in *KEAP1* at p.G333C labeled as Oncogenic by cBioPortal, was expected to have an NRF2 score surpassing the threshold as it did on project SRP066737. However, it obtained an NRF2 score of -5.40. Quality analysis via recount3 showed that this run had the fewest mapped reads both within its project (Supplementary Fig. 4a), and when compared to the A549-specific project SRP066737 (Supplementary Fig. 4b), suggesting the low count to be the reason behind the reduced accuracy of the classifier.

Other three samples with alterations in the KEAP1-NRF2 system but with low scores by our classifier were H322 (missense variant in *KEAP1* at p.R460S) with a score of −14.80, RERF-LC-MS (in-frame deletion in *KEAP1* p.G119_M120delinsV) with a score of −10.35, and H2126 (missense variant in *KEAP1* at p.R272C) with a score of −66.15. The H322 cell line, whose mutation in *KEAP1* was not listed on cBioPortal, has been reported as having low expression levels of NRF2 target genes in contrast with other KEAP1-NRF2 mutated cell lines^45^, matching the negative NRF2 score obtained for this cell line. Similarly, the in-frame deletion of RERF-LC-MS cell line is not reported on cBioPortal, and therefore the low NRF2 score might be informative of the lack of effect of this deletion on the functionality of *KEAP1*. As for the cell line H2126, our classifier failed to align the NRF2 score with the Oncogenic missense variant alteration reported on cBioPortal, resulting in what could be considered as a false negative. Further investigation is required to determine whether this outcome accurately reflects the KEAP1-NRF2 pathway dynamics in this cell line or a misclassification.

Among the positive NRF2 scores obtained for the project DRP001919, three cell lines stood out: II-18, H1437, and PC-9 with NRF2 scores of 118.16, 64.75 and 81.76 respectively (Fig. 5a). II-18 and H1437 cell lines have been previously reported as having low KEAP1 expression levels, with no documented genomic alterations^46,47^. However, our in-depth read coverage analysis and visualization using the Integrative Genomics Viewer (IGV) uncovered significant findings: a complete deletion of *KEAP1* in the II-18 line (Fig. 5b) and a deletion encompassing the promoter region, exon 1, and exon 2 of *KEAP1* in the H1437 line (Fig. 5c). Additionally, examining the coverage in the *NFE2L2* region revealed a peak indicating an *NFE2L2* amplification in the cell line II-18 (Fig. 5d). Therefore, we concluded that the particularly high NRF2 score in the II-18 cell line could be attributed to a combination of both *KEAP1* deletion and *NFE2L2* amplification.

PC-9, another cell line derived from lung adenocarcinoma studied on this project, has been reported as having high NRF2 expression^48^. Indeed, in addition to DRP001919, we found NRF2 scores surpassing our threshold in another 37 runs across 8 SRA projects involving PC-9, indicating the potential presence of an alteration in this cell line (Supplementary Data 1). However, no reports of mutation status on the KEAP1-NRF2 system were found for this cell line. Local exploration of the coverage showed no alterations, neither in *KEAP1* nor *NFE2L2*. However, more extensive exploration of the cell line showed that the scope of the *NFE2L2* amplification covers more than just the locality as it happened on the local deletions observed in II-18 and H1437. Structural variation analysis of PC-9 using the software Genomon SV from the G-CAT Workflow pipeline (see Methods) suggested a complex structural aberration on the q arm of chromosome 2 where *NFE2L2* is located (Fig. 5e). The presence of copy number alterations impacting *NFE2L2* in PC-9 cells was further supported by independent validation from the DepMap portal database (Supplementary Fig. 5).

## Discussion

In this study, we have presented a classifier based on SJ analysis for detecting constitutive activity of the KEAP1-NRF2 pathway. Our approach makes use of the subtle yet informative abnormal SJs that occur as a consequence of gene overexpression due to mutations on critical elements of the pathway, causing its dysregulation. The results obtained from the TCGA dataset support the potential of our classifier in identifying functional mutations that can impact clinical outcomes, particularly in the context of cancer. Our initial analysis focused on well-characterized mutations within the KEAP1-NRF2 pathway, outlining their prevalence across 33 cancer types and establishing a baseline for the performance of our classifier. By evaluating and integrating mutations previously labeled as Uncertain, we demonstrated the ability of the classifier to differentiate between Functional mutations, critical for cancer progression, and Non-Functional mutations, refining the predictive model. It is important to acknowledge the limitations of handling Uncertain mutations. By evaluating their scores, we were able to reclassify certain mutations as Functional, Non-Functional, or exclude them from the analysis due to inconsistent scores. Nevertheless, this approach did not differentiate between the various types of Uncertain mutations within a gene. As a result, some potentially Non-Functional mutations in *KEAP1* may have been misclassified as Functional, impacting the accuracy of our classifier. Conversely, valuable information about relevant *CUL3* mutations might have been lost due to their exclusion from the analysis. Our future research direction is to gain a deeper understanding of these individual mutations and their impact at the protein level. By utilizing advanced tools, such as deep learning models like AlphaMissense^49^, we aim to refine the classification of Uncertain mutations. Improving the distinction between Functional and Non-Functional mutations will enhance the classifier’s overall performance and lead to more reliable predictions.

Despite these challenges, the classifier demonstrated strong performance in detecting pathway activity beyond Active samples. In the evaluation of high-scoring Inactive samples, we uncovered previously undetected alterations, such as deletions and amplifications within the *KEAP1* and *NRF2* regions, which were not apparent in the initial genomic data analysis. This highlights the classifier’s utility not only as a diagnostic tool but also as a means for uncovering novel genomic insights. The discovery of such alterations on the reevaluation of the samples reinforces the importance of integrative analysis in genomic studies.

A noteworthy approach found for KEAP1-NRF2 pathway activity evaluation was the NRF2 scoring method by Härkönen et al.^21^. which involves calculating the geometric mean of the linear TMM normalized mRNA-expression of genes that form the NRF2 signature they identified. Although similar in performance to our classifier, Härkönen’s method is mainly tailored for the TCGA dataset and involves normalization specific to TCGA cancer types. In contrast, our method can be applied to various datasets and individual samples without the need for such normalization, offering broader applicability for research and clinical analysis.

Application of the classifier to the SRA database demonstrated its robustness and adaptability. The ability to discern between samples from NRF2-addicted cell lines and those with siRNA-induced suppression of NRF2 is a testament to the classifier’s specificity. Furthermore, the consistent high NRF2 scores observed in cell lines with known pathway alterations, such as Caki-2, and across diverse cancer types, highlight the versatility of our method and potential for broad application in oncological research.

However, our study is not without limitations. The anomalous NRF2 score for the A549 cell line within project DRP001919 presented a unique challenge. It revealed an important consideration for the application of our method: the quality of input data is paramount. Low counts of both mapped reads and canonical splicing counts compromised the classifier’s performance, suggesting that adequate sequencing depth is crucial for reliable predictions. This finding stresses the need for stringent quality control measures in sequencing projects to ensure data integrity.

It is important as well to clearly present the ability of our classifier in detecting both induced and constitutive activation of the pathway. While its ability to detect pathway activation is beneficial, it could lead to false positives regarding somatic mutations. Usually, oxidative stress-induced pathway activation does not involve somatic mutations in *KEAP1* or *NFE2L2*. This distinction means that although our classifier is versatile in detecting pathway activation, it may not be as precise in identifying somatic mutations, which could lead to incorrect conclusions. Therefore, researchers must carefully interpret the results to avoid potential misinterpretations.

In conclusion, our study supports the hypothesis that splicing pattern analysis is a valuable addition to the genomic toolkit for detection of abnormal pathway activity. The KEAP1-NRF2 pathway, with its pivotal role in cellular defense mechanisms, presents a prime example of how regulatory dynamics can influence gene expression and, consequently, disease pathology. Future work will focus on expanding the classifier’s application to other regulatory pathways whose dysregulation causes downstream gene overexpression, as well as exploring its potential in the context of personalized medicine. As we move towards an era of precision oncology, the integration of comprehensive genomic analyses, like the one offered by our classifier, will be crucial for the development of targeted and effective therapeutic strategies.

## Methods

### Data downloading

We applied the recount3 package to obtain SJ data from the TCGA dataset. Using the available_projects() function, we identified projects labeled with project_type = “tcga”, which yielded 10,507 tumoral samples from 33 distinct cancer types. The SJ information was downloaded by setting the type = “jxn” parameter in the create_rse() function. Through this process, we extracted three critical tables:

- An overview detailing the samples for each cancer type.
- A comprehensive list of SJ, complete with coordinates and indications of whether the junction is annotated in reference genomes.
- A matrix capturing the counts of each SJ, organized with junctions as rows and samples as columns.

The somatic mutation information as well as the clinical information with follow up were obtained from The Pan-Cancer Atlas, a comprehensive resource for cancer genomics research available at https://gdc.cancer.gov/about-data/publications/pancanatlas.

### Mutation categorization

We categorized these mutations based on their location and potential effects, as reported in scientific literature and documented in the cBioPortal database v6.0.4 (https://www.cbioportal.org/). This database classifies mutations as ‘Oncogenic’ if they are supported by scientific literature, or as ‘Likely Oncogenic’ if they are considered hotspots based purely on statistical recurrence. We downloaded mutation tables for both *KEAP1* and *NFE2L2*, using the Annotation column to select by OncoKB (oncogenicity) and CancerHotspot (defined as hotspot) keys. These labels were used to define the functionality of the mutations studied in our analysis. To obtain copy number alteration information, we gathered the masked copy number segment sets from the Genomic Data Commons data portal, accessible at https://portal.gdc.cancer.gov/. We defined CNAs as Segment_Mean values exceeding log_2_(3/2) that included the *NFE2L2* genomic coordinates in its entirety. To integrate the presence of *NFE2L2* exon 2 skipping alterations, we employed the SJ data from the TCGA dataset. We defined samples as having *NFE2L2* exon 2 skipping if they exhibited at least 2 junctions between *NFE2L2* exon 1 and exon 3, or exon 1 and exon 4 at the exon-intron boundaries. Exon boundary coordinates of exon 1, 3 and 4 for the different isoforms of *NFE2L2* were downloaded using UCSC Genome Browser tool Table Browser. The coordinates used for exon skipping labeling were 177233340, 177232584 or 177232563 (exon 3 and exon 4 start coordinates) and 177264531, 177263402, 177263436 or 177263528 (exon 1 end coordinates) of chromosome 2.

In our dataset, some samples exhibit multiple labels, indicating they possess several mutations. To improve their categorization, we ranked mutation types by their significance, assigning the highest-ranked mutation as the label of each of these samples. The hierarchy is as follows: Oncogenic *KEAP1* mutations > *NFE2L2* mutations affecting exon 2 hotspots > *KEAP1* mutations not labeled as Oncogenic > *NFE2L2* mutations affecting exon 2 on non-hotspot locations > *NFE2L2* exon 2 skipping > *CUL3* mutations > *NFE2L2* CNAs > *NFE2L2* mutations in other exons.

### Dataset refinement

Due to the lack of comprehensive data on somatic mutations and copy number alterations in all samples, our analysis used only samples with available information for these alterations. In order to also mitigate the influence of random mutations in samples with high mutation loads, we excluded 12 samples exhibiting more than 20,000 mutations. These adjustments reduced our dataset to 9533 samples.

### Selection of abnormal SJ

From the data downloaded by using recount3 and for every cancer type with a minimum of two mutated cases, we extracted abnormal SJs, which we defined as SJs not annotated in any reference genome. We then compiled a table detailing the counts of each abnormal SJ observed in the samples at least 10 times. Before statistically choosing the abnormal SJs for our classifier, we normalized each individual cancer set. To do this, we adjusted the counts of each sample: first by dividing them by the sample’s total count, and then by multiplying the result by 1,000,000. Afterwards, we further normalized the data by dividing the value of each sample by the average count of that specific abnormal SJ across the dataset. This normalization process was exclusive to the abnormal SJ selection and wasn’t applied in subsequent steps. Then, for each abnormal SJ, we conducted one-sided Wilcoxon signed-rank tests comparing the abnormal SJ counts in samples with and without alterations. The selected abnormal SJs (Bonferroni-adjusted *p*-value ≤ 0.001) obtained for each cancer type were then combined to generate the complete selection.

### Construction of classifier

Our classifier was designed based on a naive Bayes algorithm combined with a beta-binomial distribution. Specifically, we modeled the probability of a sample having the KEAP1-NRF2 system in an Active or Inactive (*m*) state (*y*) given the presence of abnormal SJs (*k*) as:

2 $${\rm{P}}(y={m|k})=\frac{{\rm{P}}({k|y}=m){\rm{P}}(y=m)}{{\rm{P}}(k)}$$

We then generated a Bayesian score representing the probability of a sample being Active given the presence of abnormal SJs, which was calculated as:

3 $${\rm{Score}}(y={m|k})=\mathrm{ln}({\rm{P}}({k|y}=m){\rm{P}}(y=m))$$

The probability distribution of our model follows a beta-binomial distribution, with the compound distribution given by:

4 $${\rm{f}}({k|n},\alpha ,\beta )=\frac{\Gamma (n+1)}{\Gamma (k+1)}\frac{\Gamma (k+\alpha )\Gamma (n-k+\beta )}{\Gamma (n+\alpha +\beta )}\frac{\Gamma (\alpha +\beta )}{\Gamma (\alpha )\Gamma (\beta )}$$

Here, *k* represents the number of reads in the feature (abnormal SJs) per sample, *n* is the total number of reads in the feature location (abnormal SJ plus normal SJ in location) per sample, and *α* and *β* are the parameters for the beta distribution. These two parameters were estimated by maximizing the log-likelihood function of the model, using a training set consisting of half the samples labeled as Active to obtain and half the samples labeled as Inactive from the TCGA dataset independently, generating two different models in the process: a model representing the distribution of abnormal SJ on Active samples and a model for Inactive samples.

In order to mitigate the impact of extreme counts in specific abnormal SJs (outliers), we implemented a threshold to limit the individual abnormal SJ scores. If the individual score of an abnormal SJ exceeds this threshold, it is truncated to match the threshold value. This limit applies to both negative and positive values. For our analysis, we set the thresholds at a value of ±10. This approach effectively controls the influence of outliers and ensures a more robust analysis of the data.

For each sample, we calculated the natural logarithm of the ratio of probabilities between the two models to determine their classification, which we refer to as the NRF2 score.

5 $$\begin{array}{l}{\rm{NRF}}2\,{\rm{Score}}={\mathrm{ln}}\left(\frac{{\rm{P}}({k|y}\,=\,{\rm{Active}}){\rm{P}}(y\,=\,{\rm{Active}})}{{\rm{P}}({k|y}\,=\,{\rm{Inactive}}){\rm{P}}(y\,=\,{\rm{Inactive}})}\right)\\\qquad\qquad\qquad ={\rm{Score}}\left(y\,=\,{\rm{Active|}}k\right)-{\rm{Score}}\left(y={\rm{Inactive|}}k\right)\end{array}$$

Since this score represents the difference in probabilities between the sample being Active and Inactive, an NRF2 score of zero can be considered as the natural threshold. A positive NRF2 score in our classifier would indicate that there is no discernible difference between the observed abnormal SJ in the sample and the abnormal SJ distribution observed across the genome of the Active samples. As a result, the sample would be classified as Active. To enhance our classifier’s robustness, we pursued a more suitable threshold. Explorations of Youden’s index^50^ as an automatic method to define the threshold retrieved negative values, which greatly differed from our decision to establish a stricter threshold than the natural zero. Ultimately, based on our independent assessment we selected a threshold of 10 as a more robust benchmark for our analysis.

### Uncertain mutations evaluation

After generating the model using the partial dataset (using Active samples and Inactive samples), our next step involved evaluating the Ambiguous samples and, based on the NRF2 score obtained, concluding whether some of these mutations should be considered Functional or Non-Functional, and therefore included in the analysis. Mutations with over 40% of instances exceeding the established threshold would be reclassified as Functional. Mutations with 40% to 10% of instances crossing the threshold would keep their Uncertain status and be subsequently removed from further analysis. Finally, mutations with less than 10% of instances exceeding the threshold would be reclassified as Non-Functional and therefore considered as not having an impact on the KEAP1-NRF2 pathway.

### Copy Number evaluation of Inactive samples with high NRF2 score

We used the masked copy number segment sets from the Genomic Data Commons data portal to extract copy number information for the *KEAP1* and *NFE2L2* genomic regions from Inactive samples exceeding the classifier threshold. The analysis was extended 15,000 bp upstream and downstream of both genes. Samples were filtered based on their Segment Mean values, selecting those with a Segment Mean ≤ −0.8 for *KEAP1* or ≥0.5 for *NFE2L2* regions. For samples with available WGS data, we conducted a detailed examination of primary alignments using IGV to identify any copy number alterations. This WGS data was obtained from the Genomic Data Commons Data Portal.

### ssGSEA analysis

For the ssGSEA analysis, we obtained expression data from the 9274 samples matching the ones used for our SJ-based classifier. To infer the mutation status of the samples based on gene expression patterns we selected the signature Human Gene Set: SINGH_NFE2L2_TARGETS, a curated collection of 14 genes associated with the NRF2 transcription factor’s targets. This gene set was obtained from the comprehensive resource jointly developed by UC San Diego and the Broad Institute, https://www.gsea-msigdb.org/.

### Precision-recall generation

In order to calculate the AUC of the Precision Recall curve, we applied the pr.curve function from the R package PRROC^51^, using as inputs the scores obtained by both our SJ-based classifier and ssGSEA analysis, as well as the mutation status associated with each sample.

### Genomic location analysis

After updating the mutation and sample classes of our dataset by the integration of Uncertain mutations, we utilized the R package GenomicRanges to analyze the overlap of our selection of 1705 abnormal SJs with coding genes. For this, we used the annotation databases generated from UCSC by using the R library TxDb.Hsapiens.UCSC.hg38.knownGene. We limit our overlap analysis to annotations whose accession numbers begin with NM (mRNA) by RefSeq.

### Complete model generation

After reassigning the labels of the mutations initially labeled as Uncertain and conducting a comparative analysis with ssGSEA, we proceeded to generate a complete model based on the entire TCGA dataset for application in independent studies such as SRA. To develop this model, we utilized every sample classified as Active or Inactive, excluding those with the final label of Ambiguous from our model’s input.

### Read coverage of bam files in *KEAP1* and *NFE2L2* regions in DRP001919

In order to investigate potentially missed copy number alterations across the genomic regions of *KEAP1* and *NFE2L2*, we explored the chromosomal read coverage of the suspected samples. Primary alignment reads with quality higher than 20 were counted on windows of 10 kb across chromosome 2 and chromosome 19 where *NFE2L2* and *KEAP1* are located respectively. Coverage was calculated by multiplying this read count by the median of read length across the region analyzed, dividing it by 10,000, and then visualized. For a more detailed visualization of the structural variations shown for PC-9, we applied the software Genomon SV from G-CAT Workflow toolkit (https://github.com/ncc-gap/GCATWorkflow) on the subset of chromosome 19 of the PC-9 bam file obtained from the DNA Data Bank of Japan Sequence Read Archive (DRA). G-CAT Workflow is a pipeline designed for cancer genome and RNA sequencing data analysis for the detection of genomic variants and transcriptomic changes. We followed its protocol and extracted the structural variation output generated by the genomonsv module which provided deletions, insertions and tandem duplications spanning the q arm of chromosome 2.

### Copy number exploration from DepMap

Copy number data for lung cell lines was downloaded from DepMap portal. Data provided is log2 of the CN ratio with a pseudo-count of 1: log_2_(Copy Number Ratio +1). The values are calculated by mapping genes to segment-level calls from data types such as whole-genome sequencing, whole exome sequencing, or SNP arrays, and then computing a weighted average along the available genomic coordinates.

### Statistics and reproducibility

The Wilcoxon test was conducted as one-tailed. For the cross-validation of our model, both the sample set with Active and Inactive sample set were divided in half. One subset from each was used for training while the other half served as the testing set, and then the roles were reversed for the second validation.

## Supplementary information

Supplementary Data 1

Supplementary Information

## Data Availability

SJ information for TCGA and SRA was obtained using the R package recount3. Copy number information was obtained from https://portal.gdc.cancer.gov/. The somatic mutation labels were obtained from https://gdc.cancer.gov/about-data/publications/pancanatlas.

## Code availability

All the code required to train the model, perform cross-validation, the SRA exploration, and the generation of the result figures is available on GitHub: https://github.com/rmateosr/SJ_Classifier_pipeline.
