# Supplementary material for: Splicing junction-based classifier for the detection of abnormal constitutive activation of the KEAP1-NRF2 system
Source: NPJ Syst Biol Appl. 2024 Dec 6;10:147. doi: 10.1038/s41540-024-00475-w (PMC11624210; doi:10.1038/s41540-024-00475-w)
Supplement: Supplementary file 2 — Supplementary Information [file 41540_2024_475_MOESM2_ESM.pdf]

Supplementary Figures:

TCGA−78−7150−01A: *KEAP1* Region

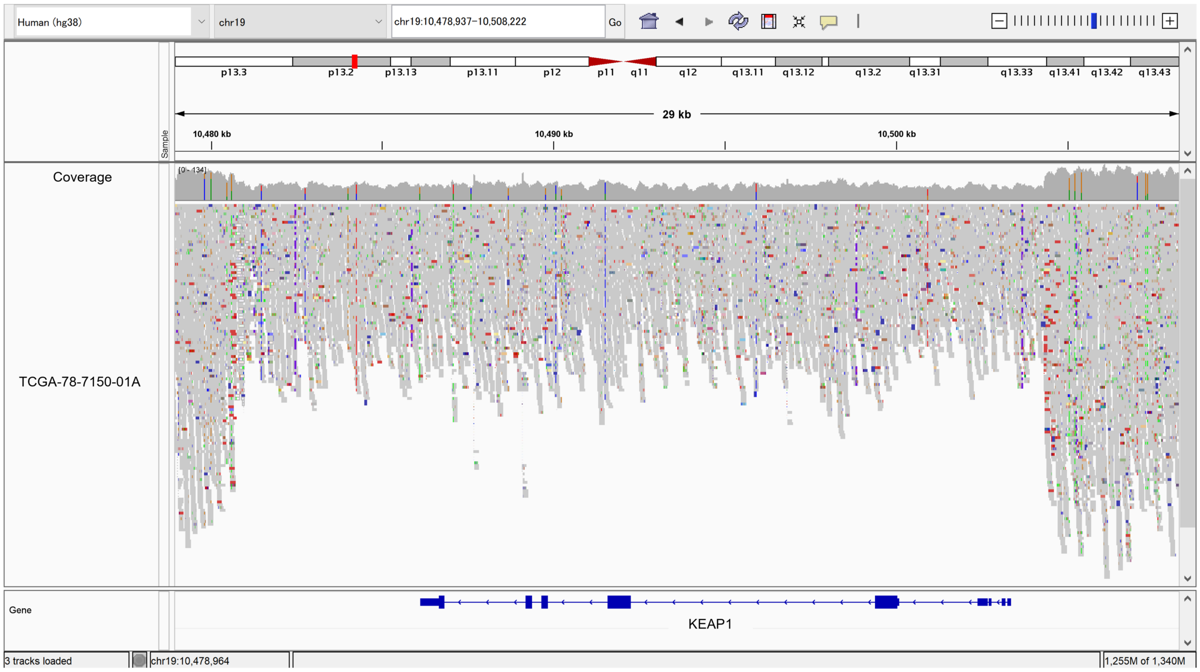

Supplementary Fig. 1. IGV visualization of the deletion of *KEAP1* in TCGA-78-7150-01A.

TCGA−DD−AACZ−01A: *KEAP1* Region

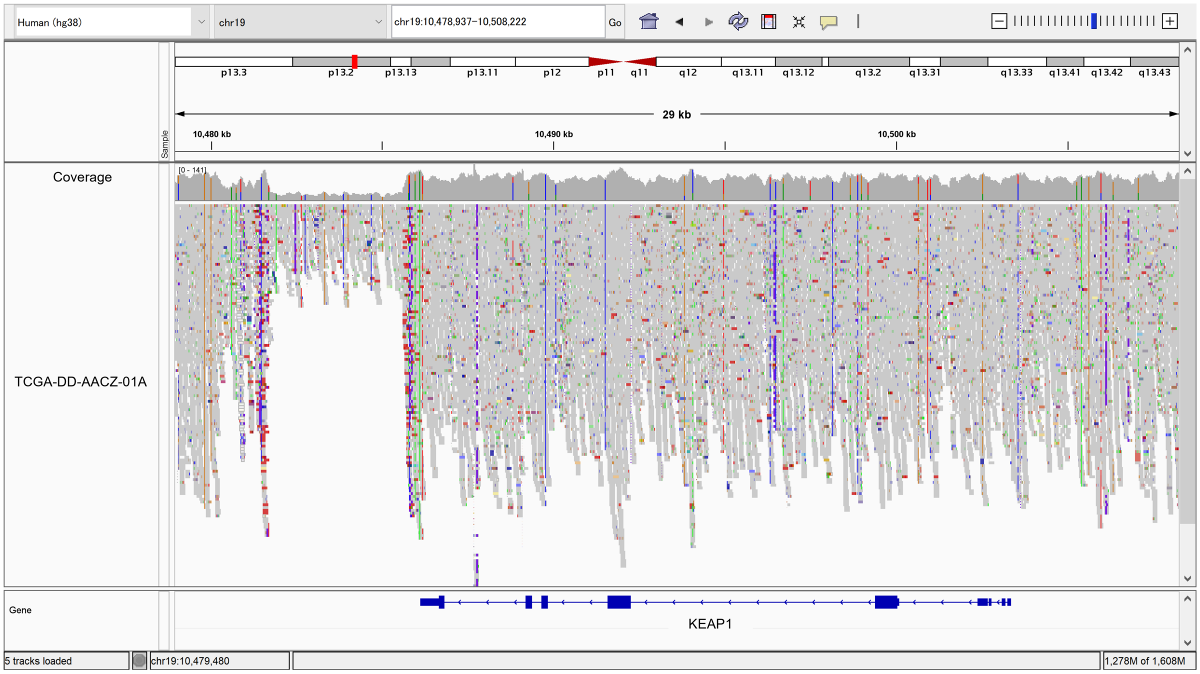

Supplementary Fig. 2. IGV visualization of the deletion downstream of *KEAP1* in TCGA-DD-AACZ-01A.

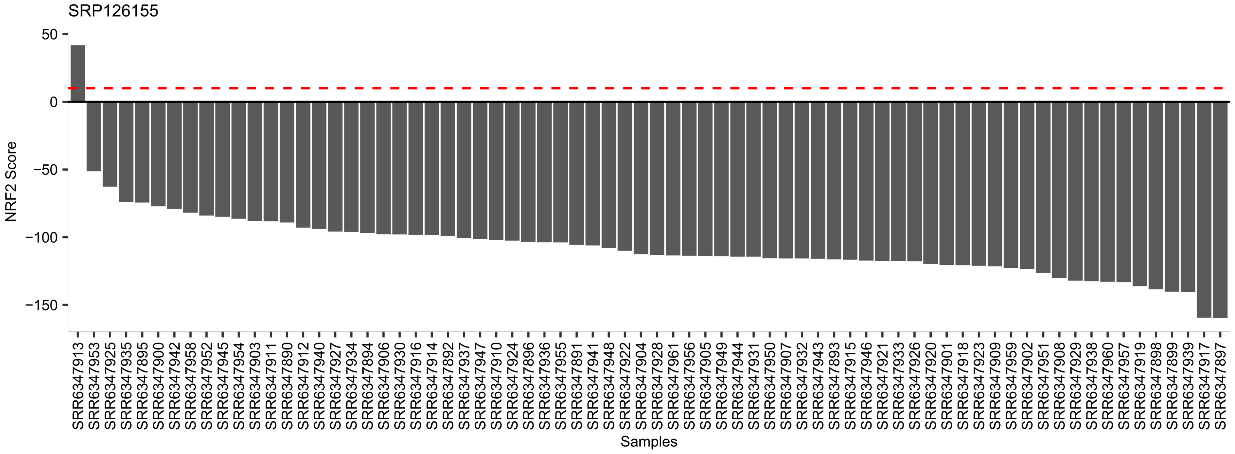

Supplementary Fig. 3. Results of our classifier on project SRP126155.

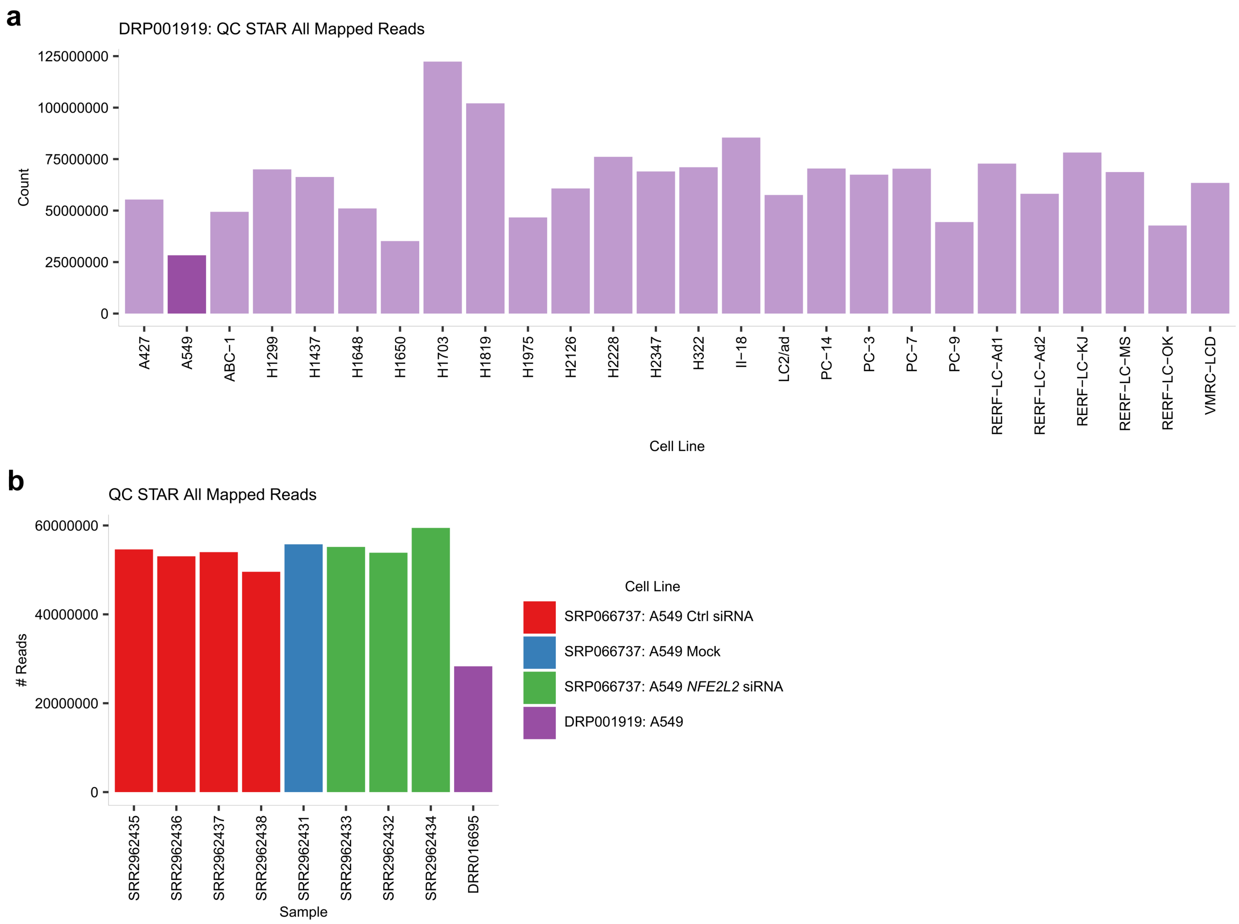

Supplementary Fig. 4. Quality assessment of A549 in DRP001919: a) DRP001919 project quality control metrics: A detailed review of recount3's quality control metrics for project DRP001919 indicates that the A549 cell line reports the lowest totals in both mapped reads and splice events. This reduced sequencing data quality likely contributed to the low NRF2 score observed for this cell line. b) SRP066737 project quality control metrics against the A549 cell line of DRP001919: A549 run from DRP001919 has also a comparably low quality when compared to an A549-focused project such as SRP066737.

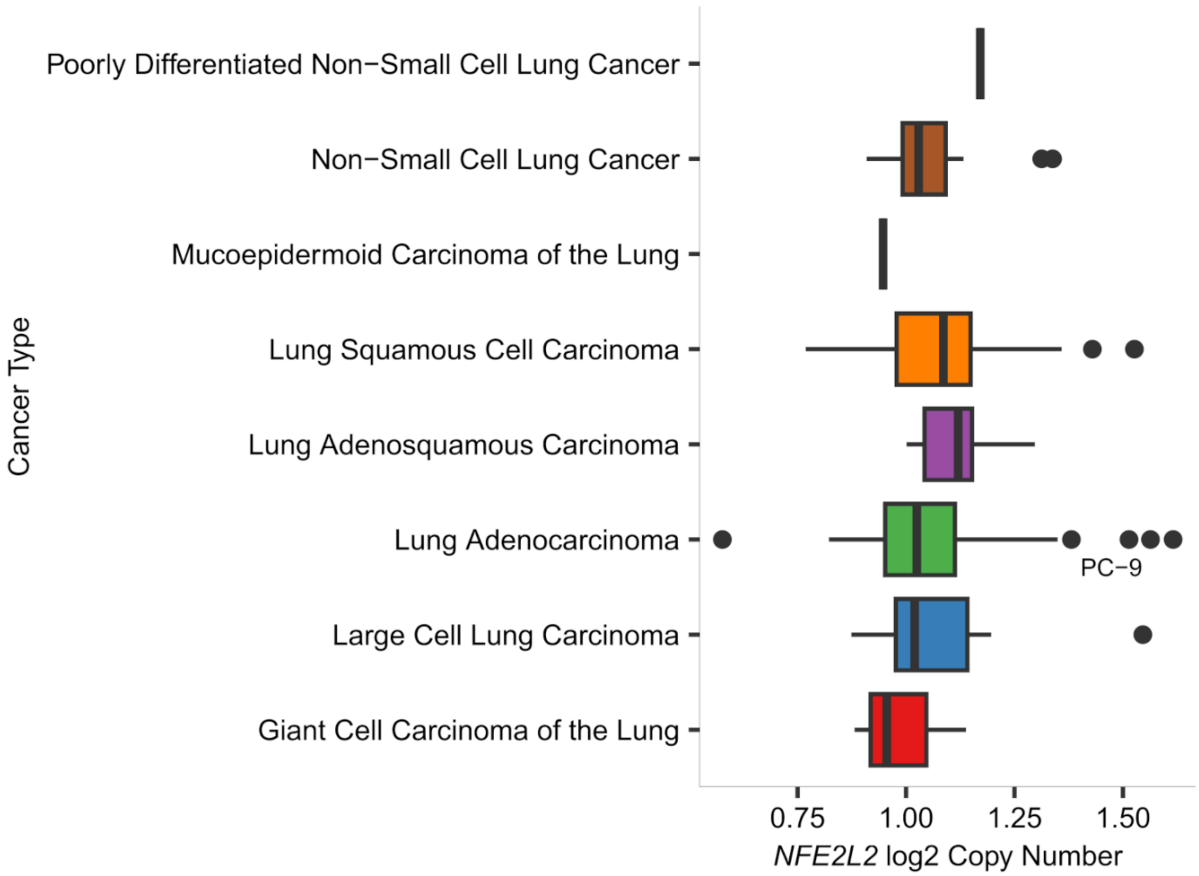

Supplementary Fig. 5. DepMap's copy number data for *NFE2L2* in lung cancer cell lines indicates an amplification in the PC-9 line, consistent with our classifier's high scoring results for this cell.

Supplementary Table 1: Report of the 15 samples with signs of potential copy number alterations in *KEAP1* and *NFE2L2* regions and with available whole genome sequencing.

TCGA_ID	Cancer	Score	Gene	Chromosome	Start	End	Num_Probes	Segment_Mean
TCGA-LN-A49U-01A	ESCA	236.6481766	KEAP1	19	283868	27749470	9059	-0.1086
TCGA-LN-A49U-01A	ESCA	236.6481766	NRF2	2	177134176	177269788	63	0.5283
TCGA-LN-A49U-01A	ESCA	236.6481766	NRF2	2	177271349	177286621	13	0.0687
TCGA-S8-A6BW-01A	ESCA	180.7643684	KEAP1	19	283868	18634853	7246	0.06
TCGA-S8-A6BW-01A	ESCA	180.7643684	NRF2	2	153924165	180396890	15879	0.541
TCGA-CV-7103-01A	HNSC	18.84691906	KEAP1	19	283868	22734049	8419	0.032
TCGA-CV-7103-01A	HNSC	18.84691906	NRF2	2	165404965	177290111	7555	0.5184
TCGA-BA-A6D8-01A	HNSC	52.62666387	KEAP1	19	283868	58370362	24162	0.1048
TCGA-BA-A6D8-01A	HNSC	52.62666387	NRF2	2	177116115	177364624	139	0.5817
TCGA-CV-6941-01A	HNSC	25.04864034	KEAP1	19	283868	27749470	9060	-0.036
TCGA-CV-6941-01A	HNSC	25.04864034	NRF2	2	177103108	177441564	189	0.5007
TCGA-CC-A5UD-01A	LIHC	34.36909766	KEAP1	19	283868	17488577	6684	-0.9396
TCGA-CC-A5UD-01A	LIHC	34.36909766	NRF2	2	58015414	241537572	97732	0.1817
TCGA-78-7150-01A	LUAD	60.27769758	KEAP1	19	283868	10478746	3525	-0.2611
TCGA-78-7150-01A	LUAD	60.27769758	KEAP1	19	10482366	10499560	9	-0.9901
TCGA-78-7150-01A	LUAD	60.27769758	KEAP1	19	10507986	10954492	192	-0.2752
TCGA-78-7150-01A	LUAD	60.27769758	NRF2	2	176949139	178412366	880	0.4401
TCGA-NC-A5HO-01A	LUSC	15.18788928	KEAP1	19	283868	58370362	23874	0.1169
TCGA-NC-A5HO-01A	LUSC	15.18788928	NRF2	2	173664309	179098548	3254	0.513
TCGA-85-6798-01A	LUSC	245.4831287	KEAP1	19	283868	23868903	9058	0.1688
TCGA-85-6798-01A	LUSC	245.4831287	NRF2	2	176732546	181187955	2792	0.5147
TCGA-94-A5I4-01A	LUSC	43.43623181	KEAP1	19	283868	21158018	7718	-0.0694
TCGA-94-A5I4-01A	LUSC	43.43623181	NRF2	2	141308625	193911165	29479	0.5001
TCGA-22-5485-01A	LUSC	516.1184957	KEAP1	19	8449396	10509520	1029	-0.0355
TCGA-22-5485-01A	LUSC	516.1184957	KEAP1	19	10510389	10510532	2	-1.7925
TCGA-22-5485-01A	LUSC	516.1184957	KEAP1	19	10513029	11053013	247	-0.0242
TCGA-22-5485-01A	LUSC	516.1184957	NRF2	2	175810466	178066361	1455	0.2018
TCGA-NK-A7XE-01A	LUSC	46.01605056	KEAP1	19	283868	23872082	9053	-0.1876
TCGA-NK-A7XE-01A	LUSC	46.01605056	NRF2	2	167202409	186514311	11336	0.5572
TCGA-LG-A6GG-01A	LIHC	22.43418151	KEAP1	19	8803416	11039529	1098	-0.8352
TCGA-LG-A6GG-01A	LIHC	22.43418151	NRF2	2	170178516	230889076	34897	0.236
TCGA-CR-7374-01A	HNSC	25.0589726	KEAP1	19	6407180	27984524	7468	0.155
TCGA-CR-7374-01A	HNSC	25.0589726	NRF2	2	175632645	178437674	1792	0.5509
TCGA-DD-AACZ-01A	LIHC	47.35058323	KEAP1	19	283868	10478746	3519	-0.263
TCGA-DD-AACZ-01A	LIHC	47.35058323	KEAP1	19	10482366	10485210	4	-1.6509
TCGA-DD-AACZ-01A	LIHC	47.35058323	KEAP1	19	10488048	23200921	5116	-0.2729
TCGA-DD-AACZ-01A	LIHC	47.35058323	NRF2	2	166146564	180617079	9164	0.106
